# Reproducibility of artificial intelligence models in computed tomography of the head: a quantitative analysis

**DOI:** 10.1186/s13244-022-01311-7

**Published:** 2022-10-27

**Authors:** Felix Gunzer, Michael Jantscher, Eva M. Hassler, Thomas Kau, Gernot Reishofer

**Affiliations:** 1 Division of Neuroradiology, Vascular and Interventional Radiology, Department of Radiology, Medical University Graz, Auenbruggerplatz 2, 8036 Graz, Austria; 2 Research Center for Data-Driven Business Big Data Analytics, Know-Center GmbH, Inffeldgasse 13/6, 8010 Graz, Austria; 3 Department of Radiology, Landeskrankenhaus Villach, Nikolaigasse 43, 9500 Villach, Austria; 4 Department of Radiology, Medical University Graz, Auenbruggerplatz 2, 8036 Graz, Austria; 5 BioTechMed Graz, Mozartgasse 12, 8010 Graz, Austria

**Keywords:** Artificial intelligence, Head CT, Reproducibility, Epidemiology, Machine learning

## Abstract

When developing artificial intelligence (AI) software for applications in radiology, the underlying research must be transferable to other real-world problems. To verify to what degree this is true, we reviewed research on AI algorithms for computed tomography of the head. A systematic review was conducted according to the preferred reporting items for systematic reviews and meta-analyses. We identified 83 articles and analyzed them in terms of transparency of data and code, pre-processing, type of algorithm, architecture, hyperparameter, performance measure, and balancing of dataset in relation to epidemiology. We also classified all articles by their main functionality (classification, detection, segmentation, prediction, triage, image reconstruction, image registration, fusion of imaging modalities). We found that only a minority of authors provided open source code (10.15%, *n* 0 7), making the replication of results difficult. Convolutional neural networks were predominantly used (32.61%, *n* = 15), whereas hyperparameters were less frequently reported (32.61%, *n* = 15). Data sets were mostly from single center sources (84.05%, *n* = 58), increasing the susceptibility of the models to bias, which increases the error rate of the models. The prevalence of brain lesions in the training (0.49 ± 0.30) and testing (0.45 ± 0.29) datasets differed from real-world epidemiology (0.21 ± 0.28), which may overestimate performances. This review highlights the need for open source code, external validation, and consideration of disease prevalence.

**Supplementary Information:**

The online version contains supplementary material available at 10.1186/s13244-022-01311-7.

## Key points

- Most research on machine learning for head CT imaging is not reproducible.
- Algorithms are not open source in most cases.
- Balancing the training data rarely mirrors real-world epidemiology.
- Graphical illustrations of model architecture were designed heterogeneously.

## Background

In the 2017 Stanford Health Trends Reports, the annual growth rate of medical data was estimated to be 48%, with 153 petabytes of data in 2013 increasing to 2314 petabytes in 2020 [1]. According to the Eurostat report on the use of imaging equipment, every 8th person in Europe needs computed tomography (CT) scan every year [2]. Diagnostic imaging accounts for a large share of these data, with increasing demands on radiologists’ daily routine. Artificial intelligence (AI), especially machine learning algorithms, will be an important tool in modern radiology to meet these demands. Machine learning is the umbrella term for computer algorithms that make accurate decisions without being programmed with explicit rules [3]. They are carried out with algorithms like convolutional neural networks (CNN), random forests (RF), support vector machines (SVM) or others [4]. However, the clinical impact of software using ML is still limited [5]. Kicky et al. [5] have shown that most products on artificial intelligence in radiology are proofs-of-concept, and few address clinical applications. To overcome this condition, applications for everyday clinical use must be developed based on sound research. There is a debate taking place that recent publications lack standardization and reproducibility [6]. Program code is rarely open source making clinical research using existing algorithms difficult, which prevents AI from being successfully implemented into healthcare environment.

In this work, we focus on algorithms using CT images of the head as training information. Image data usually goes through various pre-processing steps, and are divided into training, validation and test set. After the definition of a specific output, these sets are used as inputs for a respective algorithm. In 2020, Mongan et al. [7] noted that many publications on CNNs in the field of radiology were methodologically flawed and subsequently developed the CLAIM protocol: a checklist for research using CNNs in medical imaging. If following its rules in preparing a publication, reproducibility of the research should be ensured. In computer science, reproducibility is defined as obtaining the same results from a previous experiment under similar conditions [8]. For AI, this would be testing the same algorithm with the same hyperparameters and comparable data sets by an independent group. This term is often interchanged with replicability, which describes the simulation of a previous experiment with the same results under very different conditions by a different research group [9]. We believe that the current research lacks both, replicability, and reproducibility. In this work, we draw particular attention to the importance of providing freely accessible program codes, the significance of epidemiological factors on training data sets [10, 11], and the consistent representation of AI architectures. We set out to critically examine the current study situation based on these criteria. We analyzed research over the past twenty years in this field concerning the source of data, balancing of data sets, the reproducibility of algorithms and model performance evaluations.

## Methods

**Fig. 1:**
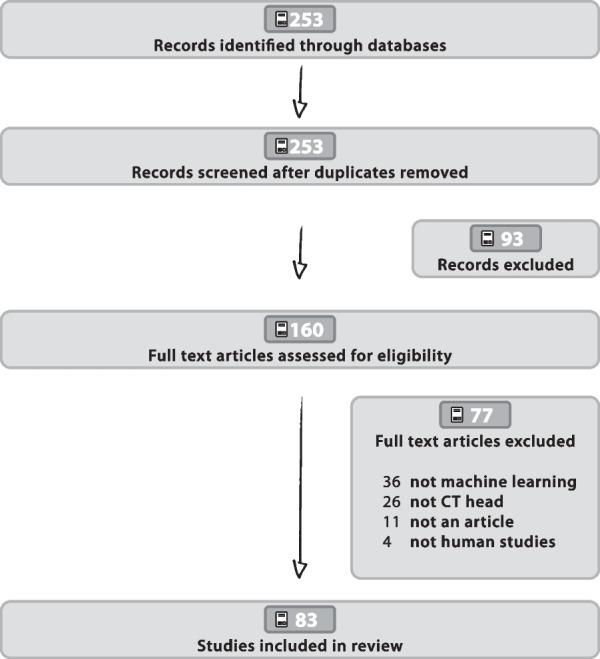
PRISMA workflow The PRISMA workflow chart explains the selection of items to be analyzed

We initially conducted a systematic literature search following the Preferred Reporting Items for Systematic Reviews and Meta-Analyses (PRISMA) guidelines [12]. The risk of bias assessment was done via the Grading of Recommendations Assessment, Development and Evaluation (GRADE) tool [13]. A PRISMA checklist can be found in the Additional file 1. This phase started on March 1, 2021, using PubMed, Cochrane, and Web of Science databases. On PubMed, we used the following Boolean search terms with Medical subjehead CTings for further stratification purposes: “(artificial intelligence [mh] OR neural network [mh] OR machine learning [mh]) & (computed tomography [mh] OR CT [mh]) & (neuroimaging [mh] OR brain imaging [mh] OR brain [mh] OR head [mh])”. We carried out two literature search phases, including articles from January 1, 2000, to December 31, 2020, and a second updated search including articles between January 1, 2021, and November 1, 2021. This period was chosen because during an initial search we recognized that the first articles on head CT relevant to this review were published after 2000 and, more importantly, only increasingly in the last 7 years. A PRISMA diagram is shown in Fig. 1.

We only selected full-length articles for review that matched the following inclusion criteria: original research article; English language; proposing a machine learning model of any kind; computed tomography of the brain; involved human participants. We preliminary excluded publications when their abstracts, read by two independent readers (G.R., F.G.), did not include research on machine learning models or papers having a SCImago Journal Rank [14] below 2, which we considered as a quality cut-off (initial screening phase). We found that an abundance of publications being published in journals below this rank does not consistently follow the suggestions for good scientific practice. We further excluded articles not involving computed tomography scans of the brain, articles not peer-reviewed or other publication types (such as letters to the editor, conference abstracts, commentaries, case reports or other systematic reviews). The collection of data and exclusion was determined by two readers (F.G., G.R.) and independently confirmed by two separate readers, one being an expert in computational science and artificial intelligence (M.J.) and one being a certified experienced radiologist (E.M.H). Machine learning was defined as a method using any sort of computer algorithm being trained with data sets to automatically solve a specific problem, in this study radiology related issues.

### Article selection

All full-length articles of the final selection (*n* = 83) were read each by one reader (F.G.), and information was manually inserted into a data frame in Excel (Office 365). A list of all variables and outcomes for which data were sought can be found in Additional file 1: Table S3. Sources of information on our epidemiological data are referenced in Additional file 1: Table S2. We analyzed research articles on algorithms with classification, detection, segmentation, or prediction tasks as main functions, where the demographic characteristics were listed, and the binary outcomes (pathology “A” or not pathology “A”) could be compared with real-word prevalence rates.

### Statistical analysis

We extracted our data into Microsoft Excel, which was then used for our statistical analysis using an R script (RStudio 4.1.1). We used descriptive statistics and a Welch Students *t*-test for the assessment of the balancing of datasets (see Table 1). Our data sets and code can be found in the repository [15] or in the Additional file 1. For the measurement of performance statistics, we classified all articles by their main functionality (classification, detection, segmentation, prediction, triage, reconstruction, generation, fusion). Features of machine learning models were considered complete when the authors defined them as satisfactory for reproducibility, meaning that the individual parameters and features were either accessible in open source code or clearly listed in publications themselves (or in the supplements or prior studies). The mere mention of parameters was not considered sufficient (e.g., "the dataset was split into a training set and a validation set" vs. "the dataset was split 80/20 into a training set and a validation set").

**Table 1:** Balancing of training and test sets compared to real world epidemiology

Data set	Mean ± SD
Training sets	0.50 + 0.31
Test sets	0.47 + 0.30
Real word epidemiology	0.22 + 0.28
*t test*	
Training sets/test sets	0.45 (.66)
Training sets/real world epidemiology	3.78 (.0004***)
Test sets/real world epidemiology	3.32 (.002**)

We statistically compared the prevalences of diseases of the training and test sets in the article selection with the respective real world epidemiology. We used a Welch Two sample *t*-test

*p < .05. **p < .01. ***p < .001; *n* = 30

### Development of research field

The literature search yielded 253 entries that met our search criteria. We excluded 93 records in our initial screening phase, keeping 160 full-text articles assessed for review. After the assessment for eligibility 83 studies were left for review. The whole selection process is shown in Fig. 1.

**Fig. 2:**
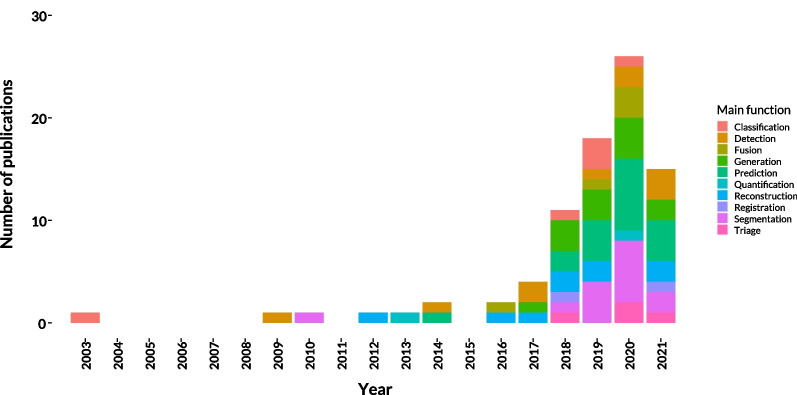
Number of publications per year and function. The number of publications started to grow around 2013 until 2020. The decrease in 2021 could be explained by the early end date of our review in November 2021 as probably not all papers of 2021 have been published yet

The number of publications has increased since 2013 with an annual growth rate of 20%. The number of publications per year starting from 2000 is shown in Fig. 2. In most studies, the main purpose of AI models was the prediction (*n* = 19) of specific events, being mostly the occurrence of intracranial lesions, outcome studies after interventions, or other pathologies concerning the brain. Other tasks included brain segmentation (*n* = 15), generation of synthetic CT images (*n* = 13), detection of lesions (*n* = 10), image reconstruction (*n* = 9), classification tasks (*n* = 8), fusion of MRI scans with CT images (*n* = 5), models on triage (*n* = 4) and automatic image registration into standardized spaces (*n* = 2).

### Transparency and SOURCES of data and code

Only a minority of authors provided open-source code (10.15%, *n* = 7). The data sets were mostly acquired from single center sources (81.9%, *n* = 68). Authors described the following steps before training as follows: augmentation steps (36.2%, *n* = 30), resolution of input scans (72.3%, *n* = 60), the definition of center of width of Hounsfield units or color space (32.5%, *n* = 27) and preprocessing steps (63.9%, *n* = 53).

### Balancing of datasets compared with epidemiology

We analyzed all articles where prevalence rates could be applied to data sets (*n* = 30). We found a mean prevalence rate used in training sets of 50% (SD ± 31%) and a mean rate of 47% (SD ± 30%) in the test sets. This differed from real-world epidemiologic data, where prevalence rates reached a mean of 22% (SD ± 28%). The balancing of training and test sets compared to real word epidemiology is shown in Table 1. References for the epidemiological data used for the calculations are listed in Additional file 1: Table S3.

### Types of algorithms

Types of machine learning algorithms used on CT brain images are (in descending order) convolutional neural networks (CNN) (*n* = 60), random forests (RF) (*n* = 8), dictionary learning (DL) (*n* = 6), support vector machines (SVM) (*n* = 6) and others (*n* = 9) (see Table 2).

**Table 2:** Frequency of algorithms used by authors

Algorithm	Frequency
CNN	60
RF	8
SVM	6
DL	6
Not defined	4
Isolation forest	1
Logistic regression	1
RBFNN	1
MLP	1
SOM	1

*CNN*, convolution neural network; *DL*, dictionary learning; *MLP*, multilayer perceptron; *RBFNN*, radial basis function neural network; *RF*, random forest; *SOM*, self organising map; *SVM*, support vector machine; *n* = 83

### Hyperparameters

As defined by J.Mongan in the CLAIM protocol [7], research papers on CNNs should at least contain the learning rate, the optimization method and the minibatch size used for training of algorithms. Furthermore, the dropout rate as well as the number of epochs should be reported, if any were used. Hyperparameters are also considered reproducible if open-source code is provided. We found the minimum number of hyperparameters defined in publications using CNNs only in eighteen cases (31.0%, *n* = 18). Authors described dropout rates for seventeen models (29.3%, *n* = 17) and defined the number of epochs in thirty-four publications (58.6%, *n* = 34).

The loss function was only defined in thirty-four cases (41.0%, *n* = 34). For the defined functions, nine used Cross Entropy loss (10.84%, *n* = 9), five Dice loss (6.0%, *n* = 5), three Mean Absolute Error loss (3.62%, *n* = 3), and two Euclidean losses (2.4%, *n* = 2). Other researchers made use of different loss functions (see Table 3).

**Table 3:** Frequency of loss functions used by authors

Loss function	Frequency
Cross entropy	9
Dice loss	5
Mean absolute error	3
Euclidean loss	2
Image gradient difference loss	1
Nesterov gradient loss	1
Stochastic gradient descent	1
Loss function described but not reproducible	13
Not defined	49

*n* = 83

### Types of networks

Pretrained networks or basic frameworks were not used or not defined in twenty-two instances (31.3%, *n* = 26). In cases where the authors defined or used already existing networks, the following were the most frequent: U-Net (20.5%, *n* = 17), ResNet (10.8%, *n* = 9), VGGNet (4.8%, *n* = 4), PItchHPERFeCT (3.6%, *n* = 3), and GoogLeNet (3.6%, *n* = 3). Ground truths were most frequently reports or decisions of radiologists (47.0%, *n* = 39), followed by raw images without any human interaction (12.1%, *n* = 10). However, a substantial portion of the authors did not define their model’s ground truth at all or not to a satisfactory extent (37.4%, *n* = 31).

### Illustration of model architectures

In sixty articles, graphical illustrations of one’s proposed model architecture were provided (72.3%, *n* = 60). The purpose of these illustrations is mostly to give readers an overview of the machine learning models. In compliance with CLAIM, the minimum details provided by these illustrations should be the data size and vector of the input data and a precise definition of the output layer. The intermediate layers may contain pooling, normalization, regularization and activation functions (again with the vector) and should show their interrelations. We propose a template of these components in the Additional file 1.

### Training and validation

The authors defined the ratio between training and validation of data sets in more than two-thirds of instances (71.1%, *n* = 59). Additionally, more than 50% (56.6%, *n* = 47) validated their predictions with a separate test set being excluded from the training or validation data. Only five authors with separate test sets used a truly external test set from a different data source (6.0%, *n* = 5).

### Metrics for model evaluation

Researchers measured a model’s sensitivity (resp. recall) and specificity, mainly in detection models (33.3%, *n* = 9; 29.6%, *n* = 8). The area under the receiver operator characteristic curve (AUROC) was predominantly used for prediction models (26.3%, *n* = 10), the dice score (resp. F1 score) mainly in segmentation applications (28.95%, *n* = 11). For models on the generation of virtual scans the mean absolute error (MAE) (22.9%, *n* = 5) and a structural similarity index (SSIM) (19.4%, *n* = 6) were primarily used as evaluation metrics. Models on fusion of imaging modalities were examined based on their peak signal-to-noise ratio (PSNR) (42.9%, *n* = 3). Additional file 1: Table S4 shows all the results in detail.

### Comparison of algorithm performances

Twenty-eight authors published their machine learning solutions as proofs-of-concept (33.7%, *n* = 28) only. Some papers let their models’ performance compete with radiologists (16.7%, *n* = 12) or other algorithms (18.1%, *n* = 18). In all other articles, the models were compared to current non-machine learning approaches.

## Discussion

With the growing number of machine learning algorithms, there is a great need for standardization of research, which is met by this review. We have carried out a detailed analysis of the reproducibility of AI research. This review focuses on the comparability of algorithms and their feasibility in the context of realism. Artificial intelligence in this particular field of radiology is one of the fastest emerging subjects, considering the number of published papers (see Fig. 2).

Only a small fraction provided open-source codes that tremendously reduce the reproducibility of research in this field being indispensable for clinical studies. Also, only a minority of publications described the necessary model’s hyperparameters to a minimum extent. Knowledge of the hyperparameters is crucial to verify the published results. This drawback can be overcome by providing open-source code, which should be available in every publication on machine learning algorithms if there is a desire to implement AI applications successfully in clinical practice.

Another major issue we found was the origin of the source data. Head CT scans were predominantly acquired at a single site. Park et al. [10] have stated that single center data lack the heterogeneity of input data for machine learning models and are, therefore, more prone to what is known as single-source bias [16]. Multicenter studies are more difficult to conduct but provide a broader range of medical data. Though a majority described the balancing of training-, validation- and test-sets, external test sets outside the training data are used only by a few. Therefore, all models with internal validation should be considered potentially inapplicable to other data. [17]. Although more challenging to establish, all machine learning models should pass external validation through data sets originating from different sources than the input data [17].

We also want to mention the important effects of epidemiological factors on the performance of AI models being rarely considered. McKnight et al. [11] have shown that machine learning algorithms perform poorly when prevalence rates drop below 10%. We found that the research papers in our review differed significantly from real-world outcome rates. The articles elaborated mainly on conditions with prevalence rates above the 10%-threshold. However, in clinical practice, pre-test probabilities below this threshold are commonly found. For test sets, the balancing should mimic the real-world occurrence of respective conditions, otherwise, a model’s evaluation of performance might be overrated, especially for rare diseases.

The majority of publications proposed algorithms for prediction, segmentation, or detection tasks. The predominant framework was that of a convolutional neural network, which was expected as CNNs provide astonishing results on object recognition tasks [18]. Only a small percentage of authors dealt with the autonomous screening and prioritization of radiological scans, namely triage, although these models might have the biggest impact on radiologists’ clinical routine in the near future [19, 20]. Binary diagnoses of lesions in the brain is an important proof-of-concept, given that imaging rates for radiologists continuously increase [21]. Particularly resource efficient handling of radiological data shall be a cornerstone of AI research. Good triage solutions might be the first to enable AI to be successfully clinically implemented.

**Fig. 3:**
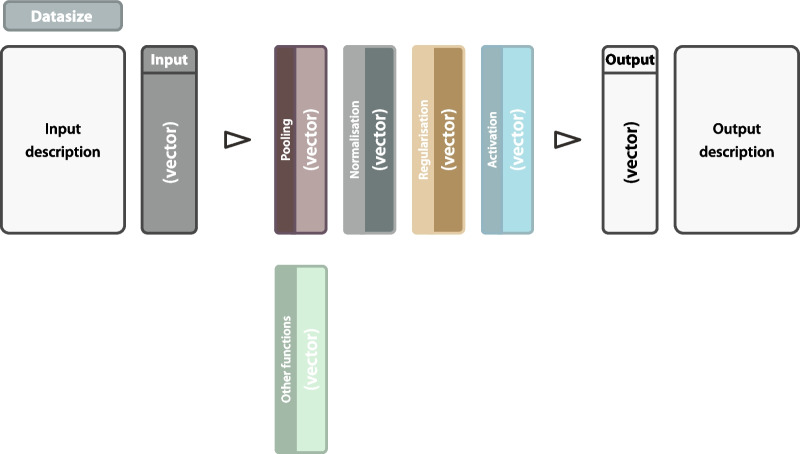
Template graphical architecture. This template incorporates all basic framework components which should be provided to readers for a quick overview. It can be used and adapted freely to one’s needs

Graphical illustrations of model architectures are tools used in the majority of publications using CNNs. The reviewed publications show a heterogeneous set of features and do not always provide readers with sufficient information. In compliance with CLAIM [7], we propose that these illustrations should at least present the input’s and output’s data size and vector, and include vectors and functions of all intermediate layers (see Fig. 3). To support the standardization of AI research, we created graphic content which can be used freely as a template [15]. Since the CLAIM protocol [7] has been published last year, the articles analyzed in our review of course did not meet its criteria. We want to encourage every author to become familiar with CLAIM and to use it as a guide for future AI publications. The results of our study must be considered cautiously as our review has several limitations. First, although we strive for completeness, there might be some articles that we did not find in the literature selection process due to unknown errors we made or unknown terminology used in studies that may have fallen through our search. Second, we only analyzed machine learning models dealing with CT imaging of the head, so our findings shall only be generalized with caution to other modalities. However, we anticipate that scientific research applying AI to other body regions or using other imaging modalities will have similar issues to those presented in this manuscript, but we cannot allege that for certain.

## Conclusions

In conclusion, current research on AI for head CT is rarely reproducible, does not match with real-world epidemiology and should be more transparent. This is essential because AI in radiology is a rapidly growing field, and therefore must meet scientific standards. We encourage scientists to consider the need for reproducibility and replicability, to standardize the representation of network architectures, and to use the CLAIM protocol [7]. It remains an open research issue whether freely available program codes and publicly reproducible algorithms significantly support the development of AI solutions, as economic considerations are not irrelevant when implementing software in healthcare providers. AI applications in radiological imaging should be considered like other diagnostic measures, whose performance is just as subject to epidemiological factors, so balancing data sets should include disease prevalence. How much epidemiology affects the performance of AI models needs to be investigated in future research.

## Supplementary Information

**Additional file 1. **Supplemental tables, PRISMA checklist and data sets used for analysis.

## Data Availability

Data generated by the authors or analyzed during the study are available at: github.com/FelixGunzer/Review_AI_CT_head. The relevant code for our statistical analysis is also publicly available at: github.com/FelixGunzer/Review_AI_CT_head.

## Abbreviations

AI: Artificial intelligence
AUROC: Area under the receiver operator characteristic curve
CNN: Convolutional neural network
DL: Dictionary learning
GRADE: Grading of recommendations assessment, development and evaluation
PRISMA: Preferred reporting items for systematic reviews and meta-analyses
PSNR: Peak signal-to-noise ratio
RF: Random forest
SVM: Support vector machin
