# Supplementary material for: Reproducibility of artificial intelligence models in computed tomography of the head: a quantitative analysis
Source: Insights Imaging. 2022 Oct 27;13:173. doi: 10.1186/s13244-022-01311-7 (PMC9613832; doi:10.1186/s13244-022-01311-7)
Supplement: Supplementary file 1 — Additional file 1. Supplemental tables, PRISMA checklist and data sets used for analysis. [file 13244_2022_1311_MOESM1_ESM.pdf]

ELECTRONIC SUPPLEMENTARY MATERIAL

Reproducibility of artificial intelligence models in computed tomography of the head – a quantitative analysis

Data files

Data generated by the authors or analysed during the study are available at:

https://github.com/FelixGunzer/Review_AI_CT_head

Tables

Table S1: List of articles

Year	Purpose	Main function	Learning type	Reference (DOI)
2003	A three-dimensional classification of brain tissue densities based on a hierarchical artificial neural network	Classification	CNN	10.1016/s0933-3657(03)00061-7
2009	Kohonen Network for automatic point correspondence of unimodal medical images	Detection	SVM	10.1016/j.compbiomed.2009.04.006
2010	The aim of this study was to derive and validate a novel, automatic, adaptive, and robust algorithm	Segmentation	-	10.1016/j.acra.2010.06.005
2012	Dictionary learning algorithm for sparse representation of given data and suggest a way to apply this algorithm to 3-D medical image denoising	Reconstruction	DL	10.1109/TBME.2011.2173935
2013	In this paper, we propose a robust sparse perfusion deconvolution method to estimate cerebral blood flow in CTP performed at low radiation dose	Quantification	DL	10.1016/j.media.2013.02.005
2014	Prediction of spontaneous intracranial haemorrhage after intervention in stroke	Prediction	SVM	10.1016/j.nicl.2014.02.003
2014	Develop an automated method for detection of the MCA dot sign of acute stroke on unenhanced CT images	Detection	SVM	10.1007/s12194-013-0234-1
2016	Image fusion method that gives the fused image better contrast, more detail information, and suitable for clinical use	Fusion	CNN	10.1007/s10278-015-9806-4
2016	Novel algorithm for MRI image reconstruction from highly undersampled MRI data and CT images	Reconstruction	DL	10.3233/XST-160540
2017	Synthetic CT Generation	Generation	CNN	10.1002/mp.12155
2017	Evaluate the performance of an artificial intelligence tool using a deep learning algorithm for detecting hemorrhage, mass effect, or hydrocephalus	Detection	CNN	10.1148/radiol.2017162664
2017	To suppress the noise effectively while retain the structures well for low dose cone beam CT image	Reconstruction	DL	10.1109/TMI.2017.2759819

Continuation of Table S1

Year	*Purpose*	*Main function*	*Learning type*	*Reference (DOI)*
2017	Radial Basis Functions Neural Network (RBFNN) based detection system, for automatic identification of Cerebral Vascular Accidents (CVA) through analysis of CT images	Detection	RBFNN	10.1016/j.cmpb.2017.05.005
2018	Stroke tissue outcome estimation	Prediction	CNN	10.3389/fneur.2018.00989
2018	Reconstruction of synthetic CTs of CSF/Brain before and after surgery	Reconstruction	DL	10.1109/TBME.2017.2783305
2018	PET attentuation correction using pseudo CTs	Reconstruction	CNN	10.1186/s40658-018-0225-8
2018	Automated deep-neural-network surveillance of cranial images for acute neurologic events	Triage	CNN	10.1038/s41591-018-0147-y
2018	Propose a novel deformable registration method, which is based on a cue-aware deep regression network, to deal with multiple databases with minimal parameter tuning	Registration	CNN	10.1109/TBME.2018.2822826
2018	This study evaluates a convolutional neural network optimized for the detection and quantification of intracranial bleeding on noncontrast CT	Segmentation	CNN	10.3174/ajnr.A5742
2018	Predict this risk of aneurysm rupture using a two-layer feed-forward artificial neural network	Prediction	CNN	10.1007/s00330-017-5300-3
2018	Deep learning angiography method to generate 3D cerebral angiograms from a single contrast-enhanced C-arm conebeam CT acquisition	Generation	CNN	10.3174/ajnr.A5597
2018	Generative adversarial approach	Generation	CNN	10.1109/TBME.2018.2814538
2018	Elucidating the relationship between the number of CT images, including data concerning the accuracy of models and contrast enhancement for classifying the images	Classification	CNN	10.1155/2018/1753480
2018	MR-to-CT synthesis task by a novel deep embedding convolutional neural network	Generation	CNN	10.1109/TBME.2018.2814538
2019	Brain haemorrhage classification	Classification	CNN	10.1155/2019/4629859
2019	Brain haemorrhage classification	Classification	CNN	10.3390/s19092167
2019	Deep learning on the brain age estimation task	Prediction	CNN	10.1016/j.mri.2019.06.018
2019	Machine learning approach to categorise the degree of collateral flow in patients who were eligible for mechanical thrombectomy and generate an e-CTA collateral score	Classification, Generation	-	10.1159/000500076
2019	Deep learning of radiological image data for outcome prediction after endovascular treatment of patients with acute ischemic stroke	Prediction	RF	10.1016/j.compbiomed.2019.103516

Continuation of Table S1

Year	*Purpose*	*Main function*	*Learning type*	*Reference (DOI)*
2019	Fusion scheme for CT and MRI medical images based on CNNs and a dual-channel spiking cortical model	Fusion	CNN	10.1007/s11517-018-1935-8
2019	Investigate the clinical utility of using CNNs to calculate ventricular volume and explore limitations	Segmentation	CNN	10.1007/s11548-019-02038-5
2019	A fully automated method that reliably computes acute intracranial lesions volume based on deep learning, cistern volume, and midline shift on noncontrast CT images	Segmentation	CNN	10.1089/neu.2018.6183
2019	Automate ASPECTS to objectively score non-contrast CTs of acute ischaemic stroke patients	Classification	RF	10.3174/ajnr.A5889
2019	A learning-based approach to improve cone beam CT's image quality for extended clinical applications	Generation	RF	10.1002/mp.13295
2019	Deep learning method to accurately reconstruct images for previously solved and unsolved CT reconstruction problems with high quantitative accuracy	Reconstruction	CNN	10.1109/TMI.2019.2910760
2019	Genetic Algorithm-based network evolution approach to search for the fittest genes to optimize network structures automatically	Reconstruction	CNN	10.1016/j.media.2019.03.004
2019	Dictionary learning-based method to segment the brain surface in post-surgical CT images of epilepsy patients following surgical implantation of electrodes	Segmentation	DL	10.1109/TMI.2018.2868045
2019	Deep-learning model trained to map projection radiographs to the corresponding 3D anatomy can subsequently generate volumetric tomographic X-ray images	Generation	CNN	10.1038/s41551-019-0466-4
2019	Machine learning-based method that evaluates large vessel occlusion and ischemic core volume in patients using CT angiography	Detection	CNN	10.1161/STROKEAHA.119.026189
2019	CNN multiplane approach and compare it to single-plane prediction of synthetic CT by using the real CT as ground truth	Prediction	CNN	10.1016/j.ijrobp.2019.06.2535
2019	Deep learning-based method to automatically segment arterio-venous malformations on CT simulation image sets	Segmentation	CNN	10.1002/mp.13560
2019	Butterfly network to perform the image domain dual material decomposition	Prediction	CNN	10.1002/mp.13489
2020	Brain haemorrhage detection and segmentation	Segmentation	CNN	10.1109/JBHI.2020.3028243

Continuation of Table S1

Year	Purpose	Main function	Learning type	Reference (DOI)
2020	Quantification of chornic ischaemic changes in acute stroke patients regarding outcome	Segmentation, Prediction	SVM	10.3389/fneur.2020.00015
2020	Classify between neoplastic and non-neoplastic intracranial haemorrhage	Prediction	RF	10.3389/fneur.2020.00285
2020	Evaluating the impact of key parameters on the pseudo CT quality generated froMRI with a 3D convolutional neural network	Classification	CNN	10.1016/j.ijrobp.2020.05.006
2020	A deep learning method applying convolutional neural networks with deep supervision for accurate hematoma segmentation and volume quantification in CT scans	Segmentation	CNN	10.1038/s41598-020-76459-7
2020	Employ machine learning algorithms to combine statistical shape information with expert ratings to generate a novel objective method of measuring the severity of metopic CS	Quantification	na	10.1097/SCS.0000000000006215
2020	Predict individual rupture status of unruptured intracranial aneurysms (UIAs)	Prediction	RF, SVM	10.1007/s00330-020-06886-7
2020	Use of deep learning techniques with CT angiography acquired from multiple medical centers and different machines to develop and evaluate an automatic detection model	Detection	CNN	10.1007/s11548-020-02121-2
2020	Medical image fusion method which combines CNNs and nonsubsampled shearlet transform (NSST) to simultaneously cover the advantages of them both	Fusion	CNN	10.1155/2020/6265708
2020	Validate a fully automated segmentation algorithm for volumetric analysis of perihaematomal edema	Segmentation	CNN	10.1161/STROKEAHA.119.026764
2020	Prediction model to identify patients in need of a non-contrast head CT exam during emergency department triage.	Triage	RF	10.1007/s00234-019-02293-y
2020	Apply deep learning methods to segment cerebral arteries on noncontrast CT scans and generate angiographies without the need for contrast administration	Segmentation, Generation	CNN	10.1371/journal.pone.0237092
2020	Deep learning method for generating virtual noncontrast CT images from contrast-enhanced CT images and evaluate its performance in dose calculations for head and neck radiotherapy	Generation	CNN	10.1002/mp.13925

Continuation of Table S1

Year	*Purpose*	*Main function*	*Learning type*	*Reference (DOI)*
2020	Use of typical networks of supervised and unsupervised deep learning methods, respectively, to transform MR/CT images to their counterpart modality	Fusion	CNN	10.1155/2020/5193707
2020	Assessing the feasibility of dose calculations from MRI acquired with a heterogeneous set of imaging protocol for paediatric patients affected by brain tumours	Generation	CNN	10.1016/j.radonc.2020.09.029
2020	Intracranial pressure based decision making: Prediction of suspected increased intracranial pressure with machine learning	Prediction	-	10.1371/journal.pone.0240845
2020	Deep learning to reliably and efficiently quantify and detect different lesion types in traumatic brain injuries	Triage	CNN	10.1016/S2589-7500(20)30085-6
2020	Detect and quantitate infarction by using non–contrast-enhanced CT scans in patients with AIS	Segmentation	CNN, RF	10.1148/radiol.2020191193
2020	A deep neural network learns to predict the final infarct volume directly from the native CTP images and metadata such as the time parameters and treatment	Classification, Prediction	CNN	10.1016/j.media.2019.101589
2020	Deep learning model to automatically detect and segment aneurysms in patients with acute subarachnoidal haemorrhage on CT angiography	Segmentation	CNN	10.1038/s41598-020-78384-1
2020	CNN to detect large vessel occlusions at multiphase CT angiography	Detection	CNN	10.1148/radiol.2020200334
2020	Examine the feasibility of deep learning techniques to convert MRI ultrashort TE images directly to synthetic CT of the skull images for use in transcranial MR imaging–guided focused ultrasound treatment planning	Generation	CNN	10.3174/ajnr.A6758
2020	Deep learning-based CNN is proposed for prediction of globe contours and their subsequent volume quantification in CT images of the orbits	Prediction	CNN	10.3174/ajnr.A6538
2020	Bidirectional convolutional LSTM (C-LSTM) network to predict 3D volumes from 4D spatiotemporal data	Prediction	CNN	10.1109/TMI.2019.2939044

Continuation of Table S1

Year	Purpose	Main function	Learning type	Reference (DOI)
2020	Combine clinical, radiomic, and quantified radiation therapy plan information for prediction of locoregional failure in head and neck cancer	Prediction	RF, CNN, Logistic Regression, Isolation Forest	10.1016/j.ejmp.2020.01.027
2020	Medical image fusion method based on the imaging characteristics of medical images	Fusion	CNN	10.1155/2020/3290136
2021	We determined the accuracy of RAPID ICH for ICH detection and ICH volumetric quantification on NCCT.	Detection	CNN	10.3174/ajnr.A6926
2021	A framework that creates synthetic data, generates ground truth data, registers multimodal images in an accurate and fast manner, and automatically classifies the image modality so that the process of registration can be fully automated.	Registration	CNN	10.1038/s41598-021-81044-7
2021	Compare the image quality of brain CT images reconstructed with deep learning–based image reconstruction and adaptive statistical iterative reconstruction-Veo.	Reconstruction	CNN	10.1007/s00234-020-02574-x
2021	Compared the added value of cuboid RF to predict the final infarct	Prediction	CNN, RF	10.1177/0271678X20924549
2021	U-net based deep learning framework to automatically detect and segment hemorrhage strokes in CT brain images.	Detection	CNN	10.1109/JBHI.2020.3028243
2021	U-Net model to perform conversions between different kV CT images	Reconstruction	CNN	10.1007/s10278-020-00414-1
2021	Deep learning algorithms to measure ventricular and cranial vault volumes in a large dataset of head CT scans	Segmentation	CNN	10.1016/j.wneu.2020.12.148
2021	Algorithm to decrease time-to-treatment, leading to improved clinical outcomes	Triage	CNN	10.1159/000515320
2021	CNN to predict the deformed left and right hemispheres	Prediction	CNN	10.1016/j.jneumeth.2020.109033
2021	Deep neural networks trained with an appropriate anatomic context in the network receptive field, can effectively perform ICH segmentation	Segmentation	CNN	10.1007/s12021-020-09493-5
2021	CNN to generate the sCT in brain site and evaluate the dosimetry accuracy	Generation	CNN	10.1002/acm2.13176
2021	Evaluate the association between perihaematomal radiomics features and haematoma expansion	Prediction	SVM	10.1016/j.crad.2021.03.003

Continuation of Table S1

Year	Purpose	Main function	Learning type	Reference (DOI)
2021	A paired–unpaired Unsupervised Attention Guided Generative Adversarial Network model to translate MR images to CT images and vice versa	Generation	CNN	10.1016/j.compbiomed.2021.104763
2021	Validation of an artificial intelligence solution for acute triage and rule-out normal of non-contrast CT head scans	Triage	CNN	10.1007/s00234-021-02826-4
2021	A radiomics-clinical model to predict intraventricular hemorrhage growth after spontaneous intracerebral hematoma	Prediction	SVM	10.18632/aging.202954

CNN - convolutional neuronal network; CT - computed tomography; CTP - computed tomography perfusion; DL - dictionary learning; MCA - middle cerebral artery; MRI - magentic resonance imaging; RF - random forest; SVM - support vector machine; n = 83

Table S2: Prevalence rates of lesions, diseases or other pathologies

Evidence	Prevalence rates			Reference (DOI)
	Training set	Test set	Real world	
Prevalence of sICH after tPA in a systematic meta-analysis	0.1415	0.1	0.06	10.1016/S0140-6736(12)60738-7
Prevlance of traumatic SAH in multicentre study of 750 patients of moderate to severe TBI	0.441	0.441	0.41	10.1097/00006123-200202000-00006
Prevalence of traumatic SDH with severe TBI	0.168	0.168	0.11	10.1227/01.Neu.0000210364.29290.C9, 10.1111/jgs.16400
Prevalence of traumatic IPH in 2875 eldery patients after fall	0.241	0.241	0.069	10.1111/jgs.16400
Prevalence of ICH in 50 patients with intracranial brain tumour	0.351	0.351	0.024	10.1007/s007010070052
Prevalence of ICB in 3088 Pat. with CT admitted to ED after mild TBI	0.377	0.415	0.048	10.1155/2013/453978
Prevlance of hydrocephalus in 2498 patients getting a CT with headaches	0.158	0.158	0.13	10.1177/1971400915602801
Prevalence of Stroke in 539 patients being admitted to Acute Stroke Unit	0.302	0.429	0.9	10.1136/emermed-2014-204392
Prevalence of ICB in 3088 Pat. with CT admitted to ED after mild TBI	0.15	0.15	0.048	10.1155/2013/453978
Prevalence of ICH in pat with haemorrhagic stroke (calculated from overall incidence of haemorrhagic stroke and ICH)	0.4	0.3	0.54	10.1016/S0733-8619(05)70200-0, 10.1016/S0140-6736(15)60692-4
Proportion of IVH in patients with haemorrhagic stroke	0.4	0.3	0.45	10.1007/s11910-010-0086-6
Prevalence of ICB in 3088 Pat. with CT admitted to ED after mild TBI	0.081	0.12	0.048	10.1155/2013/453978

Continuation of Table S2

Evidence statement	*Training set*	*Test set*	*Real world*	*Reference (DOI)*
Proportion of prevalence ounruptured intracranial aneurysms (UIA) in a systematic meta-analysis of 68 studies	0.8	0.49	0.016	10.1016/S1474-4422(11)70109-0, 10.1016/S1042-3680(18)30247-X
Prevalence of unruptured intracranial aneurysms (UIA) in a systematic meta-analysis of 68 studies	1	1	0.032	10.1016/S1474-4422(11)70109-0
Prevalence of traumatic SDH with severe TBI	0.4	0.4	0.11	10.1227/01.Neu.0000210364.29290.C9, 10.1111/jgs.16400
Prevalence of EDH in patients with TBI in a review	0.41	0.41	0.04	10.1227/01.Neu.0000210364.29290.C9
Prevalence of ICB in 3088 Pat. with CT admitted to ED after mild TBI	0.63	0.63	0.048	10.1155/2013/453978
Proportion of prevalence of unruptured intracranial aneurysms (UIA) in a systematic meta-analysis of 68 studies	0.09	0.09	0.016	10.1016/S1474-4422(11)70109-0, 10.1016/S1042-3680(18)30247-X
Prevalence of traumatic SDH with severe TBI	0.37	0.11	0.11	10.1227/01.Neu.0000210364.29290.C9, 10.1111/jgs.16400
Prevalence of EDH in patients with TBI in a review	0.48	0.44	0.04	10.1227/01.Neu.0000210364.29290.C9
Prevlance of traumatic SAH in multicentre study of 750 patients of moderate to severe TBI	0.74	0.61	0.41	10.1097/00006123-200202000-00006
Prevalence of traumatic IVH	0.22	0.17	0.014	10.1097/01.ta.0000218038.28064.9d
Prevalence of ICB in 3088 Pat. with CT admitted to ED after mild TBI	0.67	0.44	0.048	10.1155/2013/453978
Prevalence of Stroke in 539 patients being admitted to Acute Stroke Unit	1	1	0.9	10.1136/emermed-2014-204392
Proportion of prevalence of unruptured intracranial aneurysms (UIA) in a systematic meta-analysis of 68 studies	1	1	0.016	10.1016/S1474-4422(11)70109-0, 10.1016/S1042-3680(18)30247-X
Prevalence of Stroke in 539 patients being admitted to Acute Stroke Unit	0.754	0.754	0.9	10.1136/emermed-2014-204392
Proportion of LVO of prevalence of Stroke in 539 patients being admitted to Acute Stroke Unit	0.8	0.8	0.126	10.1093/neuros/nyz067, 10.1136/emermed-2014-204392
Proportion of LVO of prevalence of Stroke in 539 patients being admitted to Acute Stroke Unit	0.5	0.5	0.126	10.1093/neuros/nyz067, 10.1136/emermed-2014-204392
Prevalence of MCA dot sign in 100 patients with stroke	1	1	0.16	10.1161/01.STR.32.1.84
Prevalence of ICH in pat with haemorrhagic stroke (calculated from overall incidence of haemorrhagic stroke and ICH)	1	1	0.54	10.1016/S0733-8619(05)70200-0, 10.1016/S0140-6736(15)60692-4

The prevalence outcome rates described in demographic statistics for training sets, test sets or from real world epidemiology data can be found below. CT-computed tomography; ED - emergency department; EDH - epidural haemorrhage; ICB -intracranial bleeding; ICH - intracerebral haemorrhage; IPH -intraparenchymal haemorrhage; IVH - intraventricular haemorrhage; LVO - large vessel occlusion; MCA - middle cerebral artery; SAH - subarachnoidal haemorrhage; SDH - subdural haemorrhage; sICH- spontaneous intracerebral haemorrhage; TBI - traumatic brain injury; tPA - tissue plasminogen activator; UIA - unruptured intracranial aneurysm; n = 28

Table S3: List of variables and outcomes

Feature
Year
Purpose
Main function of algorithm
Graphical illustrations
Data source
Code open source
Size dataset
Dimension/Resolution Input
Colour space (Hounsfield Units)
Augmentation
Train/Validation split
Test holdout
External test set
Prevalence training set
Prevalence test set
Prevalence reality
Algorithm
Network
Epochs
Learning rate
Batch size
Dropout
Optimisation method
Loss of function
Hardware
Preprocessing steps
Ground truth
Metrics of performance
Comparison to

A list of all features for which data were sought.

Table S4: Measurements of performance in comparison to different classes of machine learning models

Measurement of performance	*Classification*	*Detection*	*Prediction*	*Segmentation*	*Class of paper* *Reconstruction*	*Triage*	*Fusion*	*Generation*	*Quantification*
**AUROC**	1 (7.14)	3 (11.11)	**10 (26.32)**	3 (7.89)	-	**3 (33.33)**	-	-	1 (25.00)
**Accuracy**	2 (14.29)	2 (7.41)	1 (2.63)	-	-	-	-	2 (6.45)	-
**Precision / PPV**	2 (14.29)	2 (7.41)	4 (10.52)	4 (10.53)	-	-	-	1 (3.23)	-
**Sensitivity / Recall**	2 (14.29)	**9 (33.33)**	5 (13.16)	**7 (18.42)**	-	2 (22.22)	-	2 (6.45)	-
**Specificity**	2 (14.29)	**8 (29.63)**	3 (7.89)	6 (15.79)	-	2 (22.22)	-	2 (6.45)	-
**NPV**	-	1 (3.70)	2 (5.26)	1 (2.63)	-	-	-	-	-
**Dice score / F1 score**	2 (14.29)	1 (3.70)	5 (13.16)	**11 (28.95)**	-	1 (11.11)	-	4 (12.90)	-
**R2**	-	-	1 (2.63)	2 (5.26)	-	-	-	-	-
**PSNR**	-	2	-	-	2 (20.00)	-	1 (14.29)	**6 (19.36)**	1 (25.00)
**SSIM**	-	-	1 (2.63)	-	2 (20.00)	-	**3 (42.86)**	-	-
**(R)MSE**	-	1 (3.70)	1 (2.63)	-	-	-	-	4 (12.90)	1 (25.00)
**MAE**	-	-	2 (5.26)	3 (7.89)	-	-	1 (14.29)	**7 (22.58)**	-
others	1 (7.14)	-	3 (7.89)	1 (2.63)	6 (60.00)	1 (11.11)	2 (28.57)	1 (3.23)	1 (25.00)

AUROC - area under the receiver operating characteristic curve; MAE - mean absolute error; others - not defined or uncommon metrics not being listed here; PPV - positive predictive value; NPV - negative predictive value; PSNR - peak signal-to-noise ratio; R2 - R-squared score; (R)MSE - (root) mean squared error; SSIM - structural similarity index; n = 83

PRISMA 2020 Checklist Page 1

Section and Topic	Item #	Checklist item	Location where item is reported
**TITLE**			
Title	1	Identify the report as a systematic review.	Title
**ABSTRACT**			
Abstract	2	See the PRISMA 2020 for Abstracts checklist.	Abstract
**INTRODUCTION**			
Rationale	3	Describe the rationale for the review in the context of existing knowledge.	pp. 1-2
Objectives	4	Provide an explicit statement of the objective(s) or question(s) the review addresses.	p. 2
**METHODS**			
Eligibility criteria	5	Specify the inclusion and exclusion criteria for the review and how studies were grouped for the syntheses.	pp.2–4
Information sources	6	Specify all databases, registers, websites, organisations, reference lists and other sources searched or consulted to identify studies. Specify the date when each source was last searched or consulted.	p.2-3
Search strategy	7	Present the full search strategies for all databases, registers and websites, including any filters and limits used.	p.2-3
Selection process	8	Specify the methods used to decide whether a study met the inclusion criteria of the review, including how many reviewers screened each record and each report retrieved, whether they worked independently, and if applicable, details of automation tools used in the process.	p.3-4
Data collection process	9	Specify the methods used to collect data from reports, including how many reviewers collected data from each report, whether they worked independently, any processes for obtaining or confirming data from study investigators, and if applicable, details of automation tools used in the process.	p.3
Data items	10a	List and define all outcomes for which data were sought. Specify whether all results that were compatible with each outcome domain in each study were sought (e.g. for all measures, time points, analyses), and if not, the methods used to decide which results to collect.	Supplements
	10b	List and define all other variables for which data were sought (e.g. participant and intervention characteristics, funding sources). Describe any assumptions made about any missing or unclear information.	Supplements
Study risk of bias assessment	11	Specify the methods used to assess risk of bias in the included studies, including details of the tool(s) used, how many reviewers assessed each study and whether they worked independently, and if applicable, details of automation tools used in the process.	GRADE
Effect measures	12	Specify for each outcome the effect measure(s) (e.g. risk ratio, mean difference) used in the synthesis or presentation of results.	p.GRADE
Synthesis methods	13a	Describe the processes used to decide which studies were eligible for each synthesis (e.g. tabulating the study intervention characteristics and comparing against the planned groups for each synthesis (item #5)).	pp.3-4
	13b	Describe any methods required to prepare the data for presentation or synthesis, such as handling of missing summary statistics, or data conversions.	R script
	13c	Describe any methods used to tabulate or visually display results of individual studies and syntheses.	R script
	13d	Describe any methods used to synthesize results and provide a rationale for the choice(s). If meta-analysis was performed, describe the model(s), method(s) to identify the presence and extent of statistical heterogeneity, and software package(s) used.	R script
	13e	Describe any methods used to explore possible causes of heterogeneity among study results (e.g. subgroup analysis, meta-regression).	R script
	13f	Describe any sensitivity analyses conducted to assess robustness of the synthesized results.	R script
Reporting bias assessment	14	Describe any methods used to assess risk of bias due to missing results in a synthesis (arising from reporting biases).	GRADE
Certainty assessment	15	Describe any methods used to assess certainty (or confidence) in the body of evidence for an outcome.	GRADE

Section and Topic	Item #	Checklist item	Location where item is reported
**RESULTS**			
Study selection	16a	Describe the results of the search and selection process, from the number of records identified in the search to the number of studies included in the review, ideally using a flow diagram.	Figure 1
	16b	Cite studies that might appear to meet the inclusion criteria, but which were excluded, and explain why they were excluded.	Figure 1
Study characteristics	17	Cite each included study and present its characteristics.	Supplementals
Risk of bias in studies	18	Present assessments of risk of bias for each included study.	GRADE
Results of individual studies	19	For all outcomes, present, for each study: (a) summary statistics for each group (where appropriate) and (b) an effect estimate and its precision (e.g. confidence/credible interval), ideally using structured tables or plots.	pp. 4-6
Results of syntheses	20a	For each synthesis, briefly summarise the characteristics and risk of bias among contributing studies.	GRADE
	20b	Present results of all statistical syntheses conducted. If meta-analysis was done, present for each the summary estimate and its precision (e.g. confidence/credible interval) and measures of statistical heterogeneity. If comparing groups, describe the direction of the effect.	pp.4-6
	20c	Present results of all investigations of possible causes of heterogeneity among study results.	GRADE
	20d	Present results of all sensitivity analyses conducted to assess the robustness of the synthesized results.	GRADE
Reporting biases	21	Present assessments of risk of bias due to missing results (arising from reporting biases) for each synthesis assessed.	GRADE
Certainty of evidence	22	Present assessments of certainty (or confidence) in the body of evidence for each outcome assessed.	GRADE
**DISCUSSION**			
Discussion	23a	Provide a general interpretation of the results in the context of other evidence.	pp. 7-8
	23b	Discuss any limitations of the evidence included in the review.	p. 8
	23c	Discuss any limitations of the review processes used.	p. 8
	23d	Discuss implications of the results for practice, policy, and future research.	p. 8
**OTHER INFORMATION**			
Registration and protocol	24a	Provide registration information for the review, including register name and registration number, or state that the review was not registered.	p.2
	24b	Indicate where the review protocol can be accessed, or state that a protocol was not prepared.	p.2-3
	24c	Describe and explain any amendments to information provided at registration or in the protocol.	p.2-3
Support	25	Describe sources of financial or non-financial support for the review, and the role of the funders or sponsors in the review.	p. 9
Competing interests	26	Declare any competing interests of review authors.	p. 9
Availability of data, code and other materials	27	Report which of the following are publicly available and where they can be found: template data collection forms; data extracted from included studies; data used for all analyses; analytic code; any other materials used in the review.	p. 9

From: Page MJ, McKenzie JE, Bossuyt PM, Boutron I, Hoffmann TC, Mulrow CD, et al. The PRISMA 2020 statement: an updated guideline for reporting systematic reviews. BMJ 2021;372:n71. doi: 10.1136/bmj.n71

For more information, visit: http://www.prisma-statement.org/

PRISMA checklist page 2
